# Primary Pleural Epithelioid Angiosarcoma With Extensive and Rapid Metastasis to Brain and Bilateral Adrenal Glands

**DOI:** 10.7759/cureus.9982

**Published:** 2020-08-24

**Authors:** Margaret A Sinkler, Asad Ullah, Chase J Wehrle, Muaz A Ibrahim, Joseph White

**Affiliations:** 1 Pathology, Medical College of Georgia - Augusta University, Augusta, USA; 2 Radiology, Medical College of Georgia - Augusta University, Augusta, USA

**Keywords:** pleural, adenopathy, angiosarcoma, adrenal, metastasis

## Abstract

A 64-year-old male presents with shoulder pain, arm pain, and a chronic cough. CT imaging of the thorax shows a large 8.0 x 6.7 cm mass with central necrosis in the left upper lung lobe with invasion into the chest wall with partial destruction of the second and third ribs, and left axillary adenopathy. Bilateral adrenal nodules are identified via CT imaging and subsequently biopsied. Histologically, the mass reveals sheets of atypical epithelioid cells with round nuclei and abundant eosinophilic cytoplasm. Immunostaining is positive for CD31, CD34, FLI-1, AE1/AE3, and CK7, diagnostic of primary epithelioid angiosarcoma. The patient developed symptoms of confusion, dizziness, and ataxia. An MRI showed metastatic brain lesions. One month later, the patient had worsening symptoms. Repeat imaging demonstrates enlargement of the bilateral adrenal masses, a new lesion posterior to the left kidney, and doubling of the size of the brain lesions.

This case illustrates the metastatic potential and pattern of the spread of an aggressive primary pleural angiosarcoma that is not described elsewhere in current literature. It also highlights the importance of timely intervention based on the rapid metastatic progression of this neoplasm.

## Introduction

Angiosarcomas represent only 1% of all soft tissue malignancies [1,2]. Tumor arises from the endothelial cells of blood vessels. Frequently involved sites include skin, deep soft tissue, breast, liver, bone, and spleen [1]. To the best of our knowledge, primary pleural angiosarcomas are rare and aggressive with less than 30 cases reported in English literature [3]. Most pleural neoplasms identified are metastatic. The two reported primary pleural malignant vascular tumors include epithelioid hemangioendothelioma and angiosarcoma [1]. 

The pathogenesis of primary pleural angiosarcoma is unclear. Conditions currently linked to the development of pleural epithelioid angiosarcomas include a history of chronic tuberculous pyothorax, prior radiotherapy, and asbestos exposure. The common initial clinical presentation is characterized by effusion, pleural thickening, and chest pain. Less common presentations can include bloody effusion, hemoptysis, or solitary pleural mass [1]. Frequently, symptoms present before a mass is present making initial diagnosis difficult [4]. The neoplasm is very aggressive with most patients dying within six months of diagnosis with increased mortality in patients with advanced age, site of its involvement, necrosis, and epithelioid features [1]. Treatment strategies include tumor resection, extrapleural pneumonectomy, and chemotherapy. Due to the rarity of the disease, a definitive treatment strategy has not been established. 

We present a case of a 64-year-old male who presented with shoulder pain and a chronic cough. Extensive workup and immunohistochemistry revealed the diagnosis of a primary epithelioid angiosarcoma. The patient had extensive metastasis to the brain and adrenal glands. Due to the aggressive nature of the primary neoplasm, few reports of the metastatic pattern are reported. 

## Case presentation

**Figure 1:**
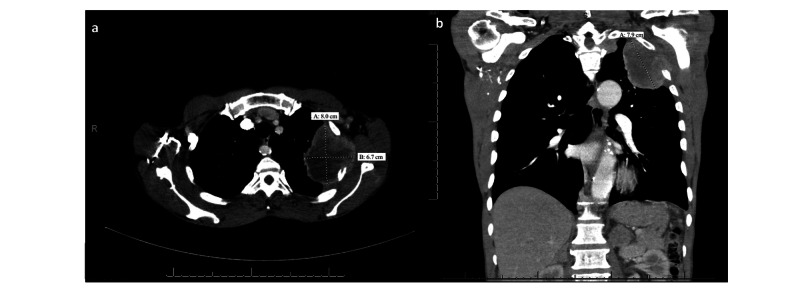
Axial and coronal CT images of the chest showing heterogeneous left apical mass Axial (a) and coronal (b) CT images of the chest demonstrating an 8.0 x 6.7 x 7.9 cm heterogeneous left apical mass with central necrosis and invasion into the left extra pleural space and chest wall with partial destruction of the second and third ribs.

**Figure 2:**
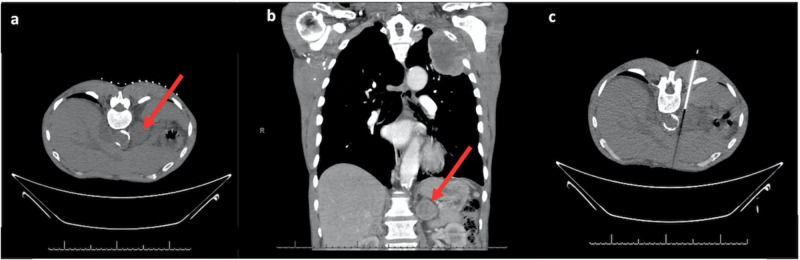
CT images of the chest showing heterogeneous enlarged left adrenal mass concerning for metastatic lesion Axial (a) and coronal (b) CT images of the chest demonstrating a heterogeneous enlarged left adrenal mass concerning for metastatic lesion (arrow). (c) Axial image obtained during CT-guided biopsy for tissue sampling.

A 64-year-old male with a past medical history of hypertension and 30 pack-year history of smoking presented to the emergency department with complaints of shoulder and arm pain with a chronic cough. The patient’s workup includes an initial chest x-ray, and subsequent CT thorax showing a large 8.0 x 6.7 cm mass in the left upper lung with central necrosis and direct invasion of the left chest wall with partial destruction of the second and third ribs with left axillary adenopathy (Figure 1). The mass had obtuse margins with the chest wall, pleural nodularity at the left lung apex, and local lymphadenopathy, which is seen with pleural-based neoplasms. Imaging also revealed bilateral adrenal nodules. CT-guided biopsy of the left adrenal mass was initially believed to be metastatic lung cancer (Figure 2). 

**Figure 3:**
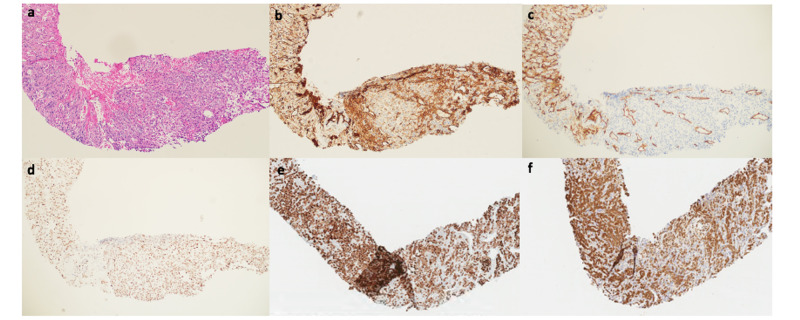
Pathologic specimen of adrenal mass (a) H&E staining shows sheets of large epithelioid cells with abundant eosinophilic cytoplasm and vesicular nuclei with moderate pleomorphism. (b) CD31 staining demonstrates membranous and cytoplasmic staining. (c) CD34 staining demonstrates strong membranous staining in tumor cells. (d) FLI1 staining shows strong nuclear staining of tumor cells. (e) CK7 shows diffuse and strong cytoplasmic staining in tumor cells. (f) AE1/AE3 shows diffuse staining of tumor cytoplasm.

Histology of the adrenal lesion revealed clusters of atypical epithelioid/polygonal cells with vesicular nuclei, prominent nucleoli, and eosinophilic cytoplasm. Immunohistochemically, tumor cells stained positive for vascular markers CD31, CD34, and FLI-1, and with keratins including CK7 and AE1/AE3 (Figure 3). Other tumors on the differential, metastatic lung cancer, mesothelioma, melanoma, and primary adrenal gland tumor, were excluded by negative staining of tumor cells for TTF-1, napsin, calretinin, WT-1, pan-melanoma, SOX10, and synaptophysin. Therefore, based on the combination of the radiology and pathology findings, the patient was diagnosed with a primary pleural angiosarcoma with early metastasis. 

**Figure 4:**
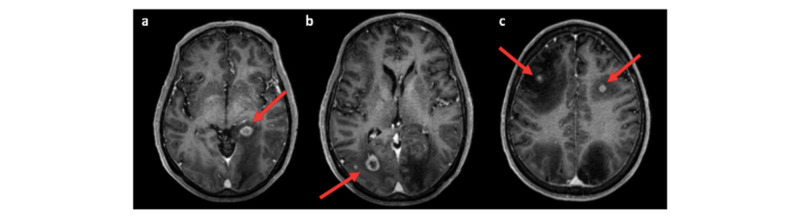
Axial T1 MRI of brain Axial post-contrast T1 MR images of the brain demonstrating ring-enhancing lesions (red arrows) in the (a) left occipitotemporal, (b) right occipital, and (c) bifrontal regions with significant surrounding vasogenic edema.

An MRI of the brain was obtained when the patient developed neurologic symptoms. A total of nine intra-axial supratentorial nodular ring-enhancing brain lesions consistent with metastases were found (Figure 4). Radiation oncology recommendations included Gamma Knife treatment of the nine metastatic brain lesions. One month after diagnosis, a repeat CT scan was obtained showing enlargement of the bilateral adrenal masses with a new 0.8 cm lesion posterior to the left kidney. A repeat brain MRI was also obtained at this time that showed a doubling of the size of the brain lesions.

Our patient was diagnosed with stage IV pleural angiosarcoma with metastases to the brain and adrenals, disqualifying the patient as a candidate for both oral and IV chemotherapy. The patient was enrolled in hospice care. 

## Discussion

The diagnosis of a primary pleural epithelioid angiosarcoma is not straightforward due to the broad differential diagnosis including mesothelioma and metastasis from poorly differentiated carcinomas from other sites. Thus, histology and immunohistochemical staining play an essential role in providing clarity to the diagnosis. 

The endothelial origin of the neoplasm can be confirmed using one of the endothelial markers (CD31, CD34, FLI-1) and epithelial markers (cytokeratins), factor VIII, and vimentin. Additional epithelial markers used for vascular tumors include CK7 and CK14. Epithelioid angiosarcomas are positive for CK8 and CK18 in roughly half the reported cases. Epithelioid variant of angiosarcoma co-expresses cytokeratin [5]. New immunomarkers such as ERG and FLI-1 have a role in cases with diagnostic difficulty. About 90% of angiosarcomas are positive for FLI-1. Histologically, pleural epithelioid angiosarcomas are characterized as infiltrative lesions with sheets of large, monomorphic oval cells with prominent nucleoli and eosinophilic cytoplasm. The combination of areas of dilated anastomotic vascular spaces with sheet-like growth, the cytomorphologic similarity between endothelial cells, and intracytoplasmic vacuolization suggest an endothelial origin for these masses [6].

Imaging studies can rarely distinguish the primary lesion from a metastatic lesion [1,7]. Our primary mass has an obtuse margin with the chest wall, nodularity of the pleura, and local lymphadenopathy. Masses of that size that originate from the lung typically create additional soft tissue nodules in the lung especially after metastasis has occurred.

Metastatic patterns of primary pleural angiosarcomas are not well documented. Pulmonary metastasis from cutaneous and cardiac angiosarcomas occurs in 60%-80% of cases [8]. Primary pleural angiosarcomas have been shown to present in most segments of the pleura with invasion commonly to the lung parenchyma, blood vessels, pericardium, diaphragm, and bronchial wall [2,4,9]. Additionally, the neoplasm has shown the potential to significantly progress. Prior reports of patients with primary pleural angiosarcomas show limited established patterns of metastasis. Patients gathered by Zhang et al. showed metastatic locations, including brain, skin, oral mucosa, and lung [10]. In their limited series, there were no reports of bilateral adrenal with brain metastasis in a single patient.

## Conclusions

Our case shows a unique pattern of metastasis and progression of an epithelioid angiosarcoma initially diagnosed from a biopsy of an adrenal mass. Histology and immunohistochemistry were diagnostic in this case. Correlation with clinical and radiologic findings supports the pleura of the lung as the primary site of this epithelioid angiosarcoma. Additionally, we report that over the course of one month metastatic lesions in the brain were significantly increased. This extensive pattern of metastasis has not been previously documented in literature. 
